# Characterization of the Major Odor-Active Compounds in Fresh Rhizomes and Leaves of *Houttuynia cordata* by Comparative Aroma Extract Dilution Analysis

**DOI:** 10.3390/foods14132303

**Published:** 2025-06-28

**Authors:** Zhenli Xu, Jing Liu, Johanna Kreissl, Claudia Oellig, Walter Vetter, Martin Steinhaus, Stephanie Frank

**Affiliations:** 1 Leibniz Institute for Food Systems Biology at the Technical University of Munich (Leibniz-LSB@TUM), Lise-Meitner-Straße 34, 85354 Freising, Germany; z.xu.leibniz-lsb@tum.de (Z.X.); j.kreissl.leibniz-lsb@tum.de (J.K.); 2 Institute of Food Chemistry, University of Hohenheim, Garbenstraße 28, 70599 Stuttgart, Germany; claudia.oellig@uni-hohenheim.de (C.O.); walter.vetter@uni-hohenheim.de (W.V.); 3 Institute of Theoretical Chemistry, Ulm University, Albert-Einstein-Allee 11, 89081 Ulm, Germany; jing-1.liu@uni-ulm.de

**Keywords:** *Houttuynia cordata*, gas chromatography–olfactometry (GC–O), comparative aroma extract dilution analysis (cAEDA), flavor dilution (FD) factor, 3-oxododecanal, (1*Z*)-1-hydroxydodec-1-en-3-one, (2*Z*)-3-hydroxydodec-2-enal, keto–enol tautomerism, Gibbs free energy

## Abstract

*Houttuynia cordata* is a culinary herb from Asia. Its edible rhizomes and leaves have a fishy aroma, the molecular background of which was unknown. A comparative aroma extract dilution analysis applied to fresh rhizomes and leaves resulted in 44 and 41 odorants, respectively, 38 of which were present with FD factors ≥1 in both samples. The odorant with the highest FD factors, whether in the rhizomes or leaves, was identified as metallic, soapy, fishy smelling 3-oxododecanal. Toward clarifying its tautomeric composition, quantum calculations suggested a predominance of the enol forms in the plant. However, the form perceived at the sniffing port during GC–O remained unclear.

## 1. Introduction

*Houttuynia cordata* is an herbaceous, flowering, and perennial plant in the family Saururaceae and native to China, Korea, Japan, and Southeast Asia [1,2]. The plant prefers warm, moist, and shady places such as fields, roadsides, and wet meadows at 300–2600 m altitude [3]. It usually grows to a 30–60 cm height and has greenish-yellow flowers, heart-shaped and reticulated veined leaves, and creeping thin rhizomes which connect the above-ground stems [2,4]. *H. cordata* is also known as chameleon, lizard’s tail, fish mint, and fish wort [5]. Some of these names refer to the characteristic fishy odor note for which the plant is known and which makes it a popular food. The main edible parts of the plant are the rhizomes and the leaves, commonly used in salads, for seasoning or stir-fried with other foods [6]. As a trendy vegetable in southwest China, its consumption history dates back to the Eastern Han dynasty (AD 25–220) and is still favored by consumers. With an average daily per capita intake of 31 g, Guiyang has the highest consumption in China [7]. In addition to its use as a foodstuff, *H. cordata* is also known as a medicinal plant with antibacterial [8,9], anti-inflammatory [10,11], antiviral [12,13], anticancer [3], antiallergic [14], antitumor [15], and antioxidative effects [10]. *H. cordata* contains many phytonutrients, e.g., flavonoids, polyphenols, steroids, and alkaloids [3,16,17,18]. The health-promoting aspect of the plant has already been studied in detail, but despite its popularity as a food, there is a lack of research on the compounds responsible for its characteristic aroma. The volatiles of *H. cordata* have already been well investigated, but no information on their odor activity is available. More than 90 volatiles, including mainly terpenes, aldehydes, ketones, alcohols, and esters, were identified based on experiments performed by solvent extraction [19,20], headspace–solid phase microextraction (HS–SPME) [18,19,21,22], flash evaporation [21], steam distillation [10,19,21,23,24,25], and simultaneous distillation extraction (SDE) [26] combined with gas chromatography–mass spectrometry (GC–MS). The volatiles with the highest concentrations reported were decanal [19], decan-1-ol [10], limonene [18], myrcene [10,19,20,24,25,26], (*Z*)-*β*-ocimene [18,26], 3-oxododecanal [18,21], *β*-phellandrene [26], *α*-pinene [10,18,19,25], *β*-pinene [18,19,20,24,25], terpinen-4-ol [23], tridecan-4-one [20], and undecan-2-one [10,18,19,21,22,24,26]. Only two papers differentiated between the volatile compounds in various plant parts, revealing similarities and differences between the edible parts, fresh rhizomes and leaves [18,20].

Despite the huge number of investigations on the volatiles of *H. cordata*, no systematic study has yet aimed at evaluating the contribution of individual compounds to the aroma of fresh rhizomes and leaves. In particular, none of the compounds mentioned so far could definitively explain the fishy odor note, which is highly characteristic for the *H. cordata* aroma independent of the plant origin [25,27] and harvest season [24]. Thus, the aim of the current research was to elucidate the molecular background of the aroma of fresh *H. cordata* rhizomes and leaves. Our study included the isolation of the volatile compounds of freshly harvested rhizomes and leaves using a gentle workup procedure based on solvent extraction and automated solvent-assisted flavor evaporation (aSAFE) [28] and the screening for odor-active compounds by the application of a comparative aroma extract dilution analysis (cAEDA) [29].

## 2. Materials and Methods

### 2.1. Plant Materials

Rhizomes and roots of *H. cordata* were collected from the Hohenheim Gardens of the University of Hohenheim, Stuttgart, Germany, and then transplanted to the Greenhouse Laboratory Center Dürnast of the TUM Plant Technology Center (PTC), Freising, Germany. The growing conditions were maintained at 16–22 °C and 25–60% relative humidity. All analyses were performed with young and healthy rhizomes and leaves, which had been harvested on the day of the experiments and showed the characteristic aroma profiles.

### 2.2. Reference Odorants

The following reference compounds at the highest available quality were purchased from commercial suppliers: **1–3**, **7–11**, **14–19**, **21–24**, **26**, **27**, **29–31**, **33–35**, **38**, **40**, **42–44**, **46**, **47** (Merck; Darmstadt, Germany), **4** (TCI; Eschborn, Germany), **5**, **45** (Alfa Aesar; Karlsruhe, Germany), **12** (Toronto Research Chemicals; Toronto, ON, Canada), **25** (Ark Pharm; Arlington Heights, IL, USA), **28** (Chemos; Altdorf, Germany), **37** (Cayman Chemicals Company; Ann Arbor, MI, USA), **41** (Thermo Fisher Scientific; Dreieich, Germany). Compound **32** was a gift from Symrise (Holzminden, Germany). Compound **6** was synthesized according to a published procedure [30] and compound **36** was obtained as detailed below.

### 2.3. Organic Solvents

Deuterated chloroform was obtained from Merck; diethyl ether, ethyl formate, and anhydrous toluene were purchased from Thermo Fisher Scientific; acetonitrile and *n*-hexane were from VWR (Darmstadt, Germany). Dichloromethane (CLN; Langenbach, Germany) was freshly distilled through a column (120 cm × 5 cm) packed with Raschig rings before use.

### 2.4. Synthesis of 3-Oxododecanal (***36***)

The compound was synthesized using procedures from the literature [31,32] with some modifications. To a mixture of undecan-2-one (3.4 g, 20 mmol) and ethyl formate (2.2 g, 30 mmol) in anhydrous toluene (15 mL), sodium (0.46 g, 20 mmol) was added gradually and under stirring while keeping the temperature below 45 °C. After stirring overnight, the reaction mixture was washed with aqueous acetic acid (30%, *v*/*v*; 20 mL) and water (20 mL). The organic phase was dried over anhydrous sodium sulfate. The solvent was removed by rotary evaporation to obtain a yellow oil (0.85 g = 22%). The crude product was dissolved in acetonitrile and purified by preparative HPLC, as detailed in the Supplementary Materials. Compound **36** was extracted from the corresponding eluate fractions with *n*-hexane; the solvent was removed by rotary evaporation, and the neat compound was stored under an argon atmosphere. GC analyses on either a polar (DB-FFAP) or nonpolar (DB-5) column resulted in a single peak. RI (DB-FFAP): 1894, RI (DB-5): 1490; odor description (GC–O): metallic, soapy, fishy; MS (EI, 70 eV), *m*/*z* (%) 55 (9), 57 (6), 58 (10), 67 (4), 69 (5), 71 (100), 83 (7), 84 (4), 85 (6), 86 (51), 87 (5), 95 (5), 98 (7), 99 (8), 124 (5), 180 (4); MS (CI, methanol), *m*/*z* (%) 154 (3), 155 (24), 156 (2), 199 (100), 200 (14).

Enol tautomer (~93%). ^1^H-NMR (400.1 MHz, CDCl_3_, 25 °C, gs-COSY): *δ* (ppm) 7.94 (d, *J* = 4.2, 1H, H-C1), 5.55 (d, *J* = 4.2, 1H, H-C2), 2.35 (t, *J* = 7.6, 2H, H-C4), 1.63 (tt, *J* = 7.4, 2H, H-C5), 1.39–1.21 (m, 12H, H-C6, H-C7, H-C8, H-C9, H-C10, H-C11), 0.90 (t, *J* = 7.1, 3H, H-C12); ^13^C-NMR (100.6 MHz, CDCl_3_, 25 °C, gs-HSQC, gs-HMBC): *δ* (ppm) 199.9 (CO, C3), 175.7 (CO, C1), 101.7 (CH, C2), 39.5 (CH_2_, C4), 31.9 (CH_2_, C10), 29.4 (CH_2_, C8/9), 29.33 (CH_2_, C8/9), 29.26 (CH_2_, C7), 29.2 (CH_2_, C6), 25.3 (CH_2_, C5), 22.7 (CH_2_, C11), 14.1 (CH_3_, C12).

Keto tautomer (~7%). ^1^H-NMR (400.1 MHz, CDCl_3_, 25 °C, gs-COSY): *δ* (ppm) 9.84 (t, *J* = 2.6, 1H, H-C1), 3.54 (d, *J* = 2.6, 2H, H-C2), 2.52 (t, *J* = 7.4, 2H, H-C4), 1.63 (tt, *J* = 7.4, 2H, H-C5), 1.39–1.21 (m, 12H, H-C6, H-C7, H-C8, H-C9, H-C10, H-C11), 0.90 (t, *J* = 7.1, 3H, H-C12). The signals (H-C5–H-C12) of the keto tautomer coincided with those of the enol tautomer; ^13^C-NMR (100.6 MHz, CDCl_3_, 25 °C, gs-HSQC, gs-HMBC): *δ* (ppm) 204.3 (CO, C3), 196.6 (CHO, C1), 56.2 (CH_2_, C2), 44.3 (CH_2_, C4), 31.9 (CH_2_, C10), 29.4 (CH_2_, C8/9), 29.33 (CH_2_, C8/9), 29.26 (CH_2_, C7), 29.0 (CH_2_, C6), 23.3 (CH_2_, C5), 22.7 (CH_2_, C11), 14.1 (CH_3_, C12). The signals (C7–C12) of the keto tautomer coincided with those of the enol tautomer.

### 2.5. Gas Chromatography

A GC–O/FID instrument was used for GC–O analyses. GC–MS analyses were performed using three different instruments: a one-dimensional GC–MS instrument with an ion trap mass spectrometer, a two-dimensional heart-cut GC–GC–HRMS instrument with an orbitrap mass spectrometer, and a comprehensive two-dimensional GC×GC–MS instrument with a time-of-flight (TOF) mass spectrometer. Details of the individual instruments are available in the Supplementary Materials.

### 2.6. Comparative Aroma Extract Dilution Analysis (cAEDA)

Fresh rhizomes and leaves of *H. cordata* were cleaned with water and then cut into 0.5–1 cm strips with a ceramic knife within 5 min. Ten grams of either rhizomes or leaves were added to saturated aqueous calcium chloride solution (30 mL for rhizomes, 60 mL for leaves). Dichloromethane (70 mL) was added, and the mixture was homogenized with a digital high-performance dispersing instrument Ultra-Turrax T25 (IKA; Staufen, Germany) under an argon atmosphere. After stirring for 3 h, the organic phase was separated and dried over anhydrous sodium sulfate. Nonvolatiles were removed by aSAFE [28] at 40 °C using an open/closed time combination for the pneumatic valve of 0.2 s/10 s. The distillate was concentrated to 1.0 mL using a Vigreux column (60 cm × 1 cm) and a Bemelmans microdistillation device [33].

The volatile isolates of the rhizomes and leaves were stepwise diluted 1:2 with dichloromethane to obtain dilutions of 1:2, 1:4, 1:8, 1:16, 1:32, etc. Each diluted sample, as well as the undiluted sample, was subjected to GC–O analysis [29] with the FFAP column. Each odorant was assigned a flavor dilution (FD) factor, representing the dilution factor of the highest diluted sample in which the odorant was perceived at the sniffing port during GC–O [29].

### 2.7. Quantitative Olfactory Profile Analyses

Freshly harvested and chopped plant material (10 g), i.e., *H. cordata* rhizomes or leaves, were placed in cylindrical polytetrafluoroethylene (PTFE) vessels (5.7 cm height, 3.5 cm i.d., 50 mL nominal volume) with lids (Bohlender; Grünsfeld, Germany). The samples were presented to 21 trained assessors (8 males and 13 females, aged 24–60 years) in a room exclusively dedicated to sensory evaluations. The room temperature was 22 ± 2 °C. The training of the assessors included weekly sensory evaluation sessions with aqueous solutions of reference odorants. The assessors were asked to orthonasally rate the intensities of nine odor descriptors, previously defined by the assessors using free choice profiling, on a seven-point scale ranging from 0 to 3 with 0.5 increments and 0 = not detectable, 1 = weak, 2 = moderate, and 3 = strong. Each descriptor was defined by the odor of an aqueous solution containing a reference compound. The nine odor descriptors and the corresponding reference compounds were “geranium leaf” (myrcene; 0.098 μg/mL), “fishy” (3-oxododecanal; 6.7 μg/mL), “metallic” ((5*Z*)-octa-1,5-dien-3-one; 0.000047 μg/mL), “citrusy” (limonene; 1.2 μg/mL), “green, grassy” ((3*Z*)-hex-3-enal; 0.013 μg/mL), “coriander leaf” ((2*E*)-dodec-2-enal; 0.026 μg/mL), “resinous” (*α*-pinene; 3.5 μg/mL), “soapy” (decanal; 1.2 μg/mL), and “fruity” (ethyl 2-methylbutanoate; 0.00083 μg/mL). The ratings of all assessors were averaged by calculating the arithmetic mean.

### 2.8. Quantum Calculations

All quantum calculations were performed using the density functional theory implemented in the Gaussian 16 software (Gaussian; Wallingford, CT, USA). First, the geometric configurations of 3-oxododecanal tautomers in the gas phase were fully optimized with hybrid functional M062X [34] combined with basis set 6-311G(d,p) [35]. Conformational searching was performed before optimization to find all low-energy initial conformers. Frequency calculations were performed at the same computational level to verify the stability of the optimized structure and offer thermal correction to the systems. The quasi rigid-rotor harmonic oscillator (quasi-RRHO) approximations were utilized to obtain the thermal corrections by considering the low frequencies. The single-point energies (SPEs) of the optimized structures were calculated with the same functional but larger basis set def2TZVP [35] to gain higher accuracy. Standard Gibbs free energies of the tautomers in the gas phase $G_{\mathrm{gas}}^{\circ }$ were obtained by summing their SPEs with the corresponding thermal dynamic corrections using Shermo [36] after performing frequency analysis. Standard Gibbs free energies of solvation $\Delta G_{S}^{*}$ were calculated using the solvation model based on density [37]. Two different solvents were considered: water and chloroform. The geometric structures of the tautomers were reoptimized in the solvation phase using M062X/6-311G(d,p). Standard Gibbs free energies of the tautomers in the solution phase $G_{\mathrm{sol}}^{*}$ were calculated by $G_{\mathrm{sol}}^{*}$ = $G_{\mathrm{gas}}^{\circ }$ + $∆G_{S}^{*}$ + $∆G^{\circ \to *}$. $∆G^{\circ \to *}$ is the Gibbs free energy change associated with moving the solute from a gas phase standard state with the pressure of 1 atm (denoted by the superscript “°”) to a solution phase standard state with the concentration of 1 mol/L (denoted by the superscript “*”) and amounts to 1.89 kcal/mol at 298.15 K. Based on the standard Gibbs free energies in the solution phase, the Boltzmann distributions were calculated at T = 298.15 K.

## 3. Results and Discussion

### 3.1. Quantitative Olfactory Profiles

**Figure 1:**
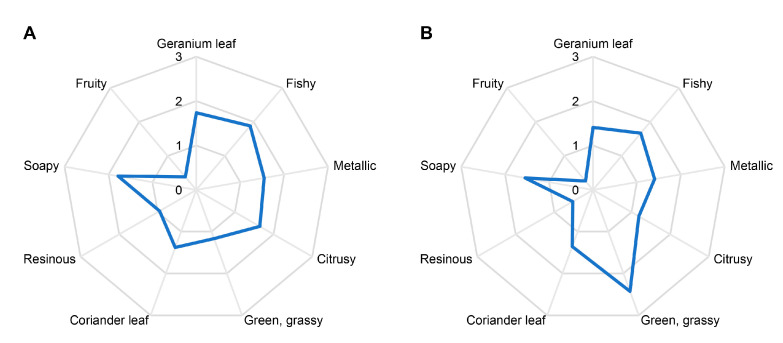
Quantitative olfactory profiles of fresh rhizomes (**A**) and leaves (**B**) of *H. cordata*. Assessors rated the intensity of each descriptor on a scale ranging from 0 to 3 with 0.5 increments and 0 = not detectable, 1 = weak, 2 = moderate, and 3 = strong.

Figure 1 shows the quantitative olfactory profiles of fresh *H. cordata* rhizomes and leaves. The profile of the rhizomes (Figure 1A) was dominated by the fishy odor note, followed by soapy, geranium leaf-like, citrusy, metallic, coriander leaf-like, and green, grassy. The green, grassy note dominated the profile of the leaves (Figure 1B). Fishy was perceived as the second most intense odor in the leaves, followed by soapy, geranium leaf-like, metallic, coriander leaf-like, and citrusy. All odor notes, except green, grassy, were rated higher in the rhizomes than in the leaves.

### 3.2. Odorant Screening

To imitate the use of *H. cordata* as a food, e.g., in salads, the freshly harvested rhizomes and leaves were chopped. This allowed for the enzymatic generation of characteristic odorants, e.g., from the lipoxygenase pathway [38,39]. After 5 min, enzymatic reactions were stopped by adding saturated aqueous calcium chloride [30]. After solvent extraction and aSAFE, the distillates were orthonasally tested on a filter paper strip to ensure successful recovery of major odorants. After evaporation of the solvent, the characteristic odor profiles of the fresh plant materials were still fully perceivable on the filter paper.

GC–O, in combination with cAEDA, applied to the volatile isolates obtained from fresh rhizomes and leaves, resulted in 44 and 41 odorants, respectively, 38 of which were present with FD factors ≥1 in both samples (Table 1). FD factors ranged up to 16,384 for the rhizomes and up to 4096 for the leaves. In both samples, the rhizomes and the leaves, the compound with the highest FD factors was metallic, soapy, fishy smelling odorant **36**. The compound clearly resembled the dominating fishy odor note in the olfactory profile of the rhizomes. In the rhizomes, high FD factors were further determined for odorant **7** (geranium leaf-like; FD factor 2048), odorant **12** (metallic; FD factor 512), odorant **2** (resinous; FD factor 256), odorant **8** (citrusy; FD factor 256), and odorant **35** (coriander leaf-like; FD factor 128). In the leaves, in addition to odorant **36**, seven more odorants showed FD factors ≥128, including odorant **7** (geranium leaf-like; FD factor 2048), odorant **12** (metallic; FD factor 1024), odorant **6** (green, grassy; FD factor 256), odorant **17** (soapy, citrusy; FD factor 256), odorant **2** (resinous; FD factor 128), odorant **11** (mushroom-like; FD factor 128), and odorant **35** (coriander leaf-like; FD factor 128).

### 3.3. Structure Assignment

The RIs obtained with two GC columns of different polarity (DB-FFAP and DB-5) for the individual *H. cordata* rhizome and leaf odorants, as well as the corresponding odor descriptions, were compared to published data, foremost those compiled in the Leibniz-LSB@TUM Odorant Database [40]. In the case of matching data, authentic reference compounds were purchased or synthesized and analyzed in an appropriate dilution by GC–O in parallel with the plant volatile isolates. The structure proposals were confirmed by comparing the mass spectra of the *H. cordata* odorants and the authentic reference compounds obtained by GC–MS analyses. In the case of coelution problems during one-dimensional GC–MS analysis, the comprehensive two-dimensional GC×GC–MS instrument was employed to obtain mass spectra without interferences. As a result, the structures of 43 odorants were successfully assigned, and only compounds **13**, **20**, **36**, and **39** remained unknown (cf. Table 1).

**Figure 2:**
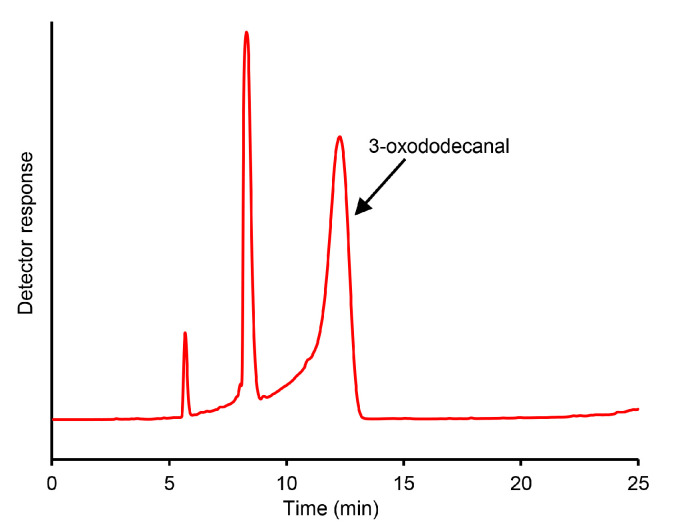
Preparative HPLC–UV chromatogram of the raw product obtained in the 3-oxododecanal synthesis.

Given the high FD factors of compound **36** in combination with its characteristic fishy odor, further structure assignment efforts were focused on this compound. Comparing its mass spectra (EI and CI) obtained by GC–MS analyses with the published literature [41] resulted in a high matching factor for 3-oxododecanal. After synthesizing 3-oxododecanal from undecan-2-one and ethyl formate and purifying the raw product by preparative HPLC–UV (Figure 2), analyses of the synthesized compound by GC–O and GC–MS on the two separation systems resulted in the same data as obtained for odorant **36**. To our knowledge, it was the first time that the olfactory potential of 3-oxododecanal was recognized. The presence of 3-oxododecanal in *H. cordata* has already been mentioned in the literature [18,21], but its potential contribution to the plant’s characteristic odor has not yet been considered.

High FD factors (≥128) in the rhizomes were additionally assigned to odorants **7**, **12**, **2**, **8**, and **35**. They were identified as geranium leaf-like smelling myrcene (**7**; FD factor 2048), metallic smelling (5*Z*)-octa-1,5-dien-3-one (**12**; FD factor 512), resinous smelling *α*-pinene (**2**; FD factor 256), citrusy smelling limonene (**8**; FD factor 256), and coriander leaf-like smelling (2*E*)-dodec-2-enal (**35**; FD factor 128). Myrcene, *α*-pinene, and limonene have been identified in *H. cordata* before, but their aroma potency has not yet been recognized. (5*Z*)-Octa-1,5-dien-3-one and (2*E*)-dodec-2-enal have not been reported in *H. cordata*. Further odorants with comparatively high FD factors were identified as decanal (**17**; soapy, citrusy; FD factor 64), (*E*)-*β*-damascenone (**32**; cooked apple-like; FD factor 64), geraniol (**33**; rose-like, citrusy; FD factor 64), *trans*-isoeugenol (**45**; smoky, clove-like; FD factor 64), vanillin (**47**; vanilla-like; FD factor 64), (3*Z*)-hex-3-enal (**6**; green, grassy; FD factor 32), octanal (**10**; citrusy, green; FD factor 32), oct-1-en-3-one (**11**; mushroom-like; FD factor 32), 3-(methylsulfanyl)propanal (**16**; cooked potato-like; FD factor 32), undecan-2-one (**22**; soapy, green; FD factor 32), butanoic acid (**23**; sweaty; FD factor 32), 3-methylnonane-2,4-dione (**28**; hay-like, aniseed-like, fishy; FD factor 32), geranyl acetate (**31**; floral, rose-like; FD factor 32), and eugenol (**42**; clove-like; FD factor 32). Of these, (*E*)-*β*-damascenone, *trans*-isoeugenol, vanillin, octanal, oct-1-en-3-one, 3-(methylsulfanyl)propanal, butanoic acid, 3-methylnonane-2,4-dione, and eugenol have not been reported as constituents of fresh *H. cordata* rhizomes before.

In the leaves, apart from compound **36**, high FD factors (≥128) were additionally found for odorants **7**, **12**, **6**, **17**, **2**, **11**, and **35**, which were identified as geranium leaf-like smelling myrcene (**7**; FD factor 2048), metallic smelling (5*Z*)-octa-1,5-dien-3-one (**12**; FD factor 1024), green, grassy smelling (3*Z*)-hex-3-enal (**6**; FD factor 256), soapy, citrusy smelling decanal (**17**; FD factor 256), resinous smelling *α*-pinene (**2**; FD factor 128), mushroom-like smelling oct-1-en-3-one (**11**; FD factor 128), and coriander leaf-like smelling (2*E*)-dodec-2-enal (**35**; FD factor 128). Myrcene, (3*Z*)-hex-3-enal, decanal, and *α*-pinene have been previously reported in *H. cordata*, but their aroma potency has not yet been acknowledged. (5*Z*)-Octa-1,5-dien-3-one, oct-1-en-3-one, and (2*E*)-dodec-2-enal have not been identified in *H. cordata*. Further odorants with comparatively high FD factors were identified as ethyl 2-methylbutanoate (**3**; fruity; FD factor 64), 3-methylnonane-2,4-dione (**28**; hay-like, aniseed-like, fishy; FD factor 64), geranyl acetate (**31**; floral, rose-like; FD factor 64), (*E*)-*β*-damascenone (**32**; cooked apple-like; FD factor 64), octanal (**10**; citrusy, green; FD factor 32), (2*E*,4*E*)-nona-2,4-dienal (**27**; fatty, green; FD factor 32), *trans*-4,5-epoxy-(2*E*)-dec-2-enal (**37**; metallic; FD factor 32), 4-methoxybenzaldehyde (**38**; woodruff-like, aniseed-like; FD factor 32), 4-methylphenol (**40**; phenolic; FD factor 32), and *trans*-isoeugenol (**45**; smoky, clove-like; FD factor 32). Of these, only geranyl acetate and (*E*)-*β*-damascenone have been detected in fresh *H. cordata* leaves before, but their odor potential for the plant has not yet been determined.

Among the nine compounds with FD factors ≥128 in at least one of the two samples, four compounds, namely, myrcene, (5*Z*)-octa-1,5-dien-3-one, *α*-pinene, and (2*E*)-dodec-2-enal, showed comparable FD factors in both parts of the plant. Higher FD factors in the rhizomes than in the leaves were obtained for 3-oxododecanal and limonene. In contrast, higher FD factors in the leaves were found for (3*Z*)-hex-3-enal, decanal, and oct-1-en-3-one. These results were in accordance with the quantitative olfactory profiles (cf. Figure 1), where the fishy and citrusy odor intensities in fresh rhizomes (1.9 and 1.6) were higher than in leaves (1.7 and 1.2). Conversely, the intensity of the green, grassy odor note was more pronounced in the leaves (2.4) than in the rhizomes (1.2).

### 3.4. Tautomeric Distribution in ***36***

**Figure 3:**
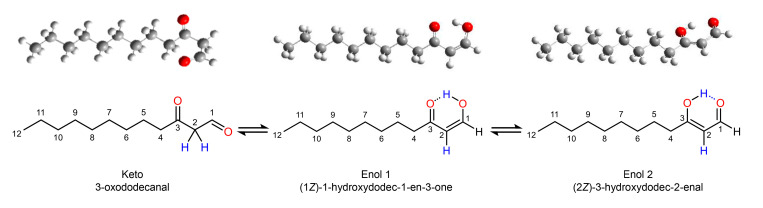
Structures of 3-oxododecanal tautomers.

With its 1,3-dicarbonyl structure, the compound can undergo keto–enol tautomerization. As a consequence, it could be present in three different forms, namely, 3-oxododecanal (keto), (1*Z*)-1-hydroxydodec-1-en-3-one (enol 1), and (2*Z*)-3-hydroxydodec-2-enal (enol 2) (Figure 3).

The ^1^H-NMR and ^13^C-NMR data of synthesized **36** obtained in deuterated chloroform (cf. Section 2) showed a predominance of one enol structure (~93%). The domination of the enol forms in deuterated chloroform has already been reported [42]. However, an organic solution does not reflect the situation at the sniffing port during GC–O or the situation in the plant, where the tautomeric distribution may be different.

GC–O analyses of synthesized **36** using either a polar or nonpolar column resulted in only a single peak with a parallel odor perception. Likewise, the mass spectra obtained by GC–MS were consistent throughout the peak. A potential explanation for these observations is a rapid interchange between the tautomers. The situation was different for 3-methylnonane-2,4-dione (**28**). GC–O analysis of the reference compound using the nonpolar DB-5 column resulted in two odor-active peaks associated with 3-methylnonane-2,4-dione tautomers. A comparison with literature data identified the first peak as the keto form and the second as the enol forms [43]. The mass spectra obtained by GC–MS confirmed the elution order. The retention and mass spectral data of hay-like smelling compound **28** perceived during the GC–O analyses of the *H. cordata* volatiles were in agreement with the first peak of the reference compound, while no odor was perceived during the elution of the second peak. Thus, compound **28** was identified as the keto tautomer 3-methylnonane-2,4-dione.

To obtain an insight into the tautomeric composition of compound **36** in the plant, quantum calculations were applied. After testing various combinations of functionals and basis sets for the standard Gibbs free energy calculation, M062X/6-311G(d,p) was selected (cf. Supplementary Materials, Table S1). The calculations were based on a single-molecule model at ambient temperature and water as the solvent. Water was chosen as the plant material contains >90% water [44]. The standard Gibbs free energies and Boltzmann distributions of the three tautomers in water are shown in Table 2. In the gas and solution phases, the enol tautomers had consistently lower Gibbs free energies than the keto tautomer, with enol 1 showing the lowest energy. According to the Boltzmann distributions, the enol forms accounted for 96% of the total composition. A higher stability of the enol tautomers was in agreement with the formation of a circular ring structure with conjugated double bonds (Figure 3, enol 1 and enol 2). Experiments and calculations with 3-oxodecanal and 3-oxobutanal resulted in quite similar tautomeric distributions [32,45].

In addition to water, Table 2 shows the standard Gibbs free energy calculations in chloroform. Both approaches with chloroform, the calculation (Table 2) and the NMR measurements (cf. above), revealed a predominance of the enol tautomers. However, the NMR experiments did not allow for a clear assignment to enol 1 or enol 2. The calculated dominance of enol 1 could not be confirmed with certainty. Nevertheless, the chemical shifts tended to favor enol 1. In summary, the quantum calculation predicted the tautomeric composition of compound **36** in the *H. cordata* plant to be mainly (1*Z*)-1-hydroxydodec-1-en-3-one and/or (2*Z*)-3-hydroxydodec-2-enal.

## 4. Conclusions

Our systematic odorant screening approach applied to fresh rhizomes and leaves of *H. cordata* resulted in 44 and 41 odorants, respectively. The odorant with the highest FD factors, whether in the rhizomes or leaves, was identified as metallic, soapy, fishy smelling 3-oxododecanal. Toward clarifying its tautomeric composition, quantum calculations suggested a predominance of the enol forms in the plant. However, the form perceived at the sniffing port during GC–O remained unclear; the experiments did not allow for the assignment of a tautomeric form to the odor activity. Additional experiments are required to identify the key odorants of fresh *H. cordata* rhizomes and leaves, and confirm the hypothesis that 3-oxododecanal is key to the characteristic fishy note. These experiments should include the quantitation of major odorants, the sensory evaluation of odor reconstitution models based on the obtained concentrations as proof of success, and omission tests to assess the contribution of the individual odorants to the overall aroma.

## Figures and Tables

**Table 1:** Odorants in the volatile isolates obtained from fresh rhizomes and leaves of *H. cordata*.

No. ^1^	Odorant ^2^	Odor ^3^	RI ^4^		FD Factor ^5^	
			FFAP	DB-5	Rhizomes	Leaves
**1**	butane-2,3-dione	butter	983	600	4	2
**2**	*α*-pinene	resinous	1024	936	256	128
**3**	ethyl 2-methylbutanoate	fruity	1047	860	2	64
**4**	2-methylpropan-1-ol	malty	1083	633	<1	2
**5**	hex-1-en-3-one	rubber, pungent	1091	788	1	2
**6**	(3*Z*)-hex-3-enal	green, grassy	1135	804	32	256
**7**	myrcene	geranium leaf	1152	1004	2048	2048
**8**	limonene	citrusy	1186	1033	256	8
**9**	*γ*-terpinene	gasoline	1233	1060	8	<1
**10**	octanal	citrusy, green	1271	1008	32	32
**11**	oct-1-en-3-one	mushroom	1286	980	32	128
**12**	(5*Z*)-octa-1,5-dien-3-one	metallic	1351	984	512	1024
**13**	unknown	fruity, honey	1374	1109	2	8
**14**	3-isopropyl-2-methoxypyrazine ^6^	earthy, pea	1407	1096	4	<1
**15**	acetic acid	vinegar	1422	638	4	2
**16**	3-(methylsulfanyl)propanal	cooked potato	1432	912	32	<1
**17**	decanal	soapy, citrusy	1478	1202	64	256
**18**	3-isobutyl-2-methoxypyrazine ^6^	green bell pepper	1500	1183	16	<1
**19**	linalool	citrusy, floral	1511	1109	1	4
**20**	unknown	herbaceous, clove	1536	1113	<1	4
**21**	bornyl acetate	mountain pine	1558	1287	16	2
**22**	undecan-2-one	soapy, green	1584	1300	32	16
**23**	butanoic acid	sweaty	1600	818	32	8
**24**	2-/3-methylbutanoic acid	sweaty	1637	870	4	2
**25**	(2*Z*)-2-butyloct-2-enal	citrusy, soapy	1658	1386	8	16
**26**	borneol	earthy, moldy	1661	1170	4	<1
**27**	(2*E*,4*E*)-nona-2,4-dienal	fatty, green	1692	1217	4	32
**28**	3-methylnonane-2,4-dione	hay, aniseed, fishy	1700	1248	32	64
**29**	pentanoic acid	sweaty	1706	908	<1	2
**30**	(*R*)-carvone	spearmint	1710	1248	16	16
**31**	geranyl acetate	floral, rose	1729	1381	32	64
**32**	(*E*)-*β*-damascenone	cooked apple	1789	1386	64	64
**33**	geraniol	rose, citrusy	1831	1261	64	4
**34**	2-methoxyphenol ^6^	smoky, gammon	1841	1088	8	4
**35**	(2*E*)-dodec-2-enal	coriander leaf	1854	1467	128	128
**36**	3-oxododecanal ^7^	metallic, soapy, fishy	1894	1490	16,384	4096
**37**	*trans*-4,5-epoxy-(2*E*)-dec-2-enal	metallic	1994	1381	16	32
**38**	4-methoxybenzaldehyde	woodruff, aniseed	2013	1257	16	32
**39**	unknown	metallic	2033	1470	16	8
**40**	4-methylphenol	phenolic	2053	1083	16	32
**41**	*γ*-decalactone	peach, coconut	2140	1467	2	<1
**42**	eugenol ^6^	clove	2153	1362	32	8
**43**	sotolon	fenugreek	2187	1108	4	4
**44**	decanoic acid	soapy, musty	2250	1381	4	8
**45**	*trans*-isoeugenol ^6^	smoky, clove	2343	1452	64	32
**46**	phenylacetic acid ^6^	honey, beeswax	2523	1274	4	4
**47**	vanillin	vanilla	2557	1405	64	16

^1^ Odorants were consecutively numbered according to their retention time on the FFAP column. ^2^ Odorants showing an FD factor of ≥1 in either of the two samples; odorants were identified by comparing their retention indices on two GC capillaries of different polarity (DB-FFAP, DB-5), their mass spectra obtained by GC–MS, and their odor quality as perceived at the sniffing port during GC–O to data obtained from authentic reference compounds analyzed under equal conditions [29]. ^3^ Odor as perceived at the sniffing port during GC–O. ^4^ Retention index; calculated from the retention time of the odorant and the retention times of adjacent *n*-alkanes by linear interpolation [29]. ^5^ Flavor dilution factor; dilution factor of the highest diluted volatile isolate in which the odorant was detected during GC–O by any of two assessors [29]. ^6^ An unequivocal mass spectrum of the compound could not be obtained; identification was based on the remaining criteria detailed in footnote 2 and by spiking experiments using GC–O/FID. ^7^ Tautomeric composition unknown.

**Table 2:** Standard Gibbs free energies (kcal/mol) and Boltzmann distributions (%) of 3-oxododecanal tautomers as obtained from quantum calculations.

**Solvent**	Tautomer	$G_{\mathrm{gas}}^{\circ }$ ^1^	$∆G_{S}^{*}$ ^2^	$G_{\mathrm{sol}}^{*}$ ^3^	$∆G_{\mathrm{sol}}^{*}$ ^4^	BD ^5^
water	enol 1	−389,465.001	−2.516	−389,465.626	1.789	82.452
	enol 2	−389,464.343	−2.102	−389,464.555	0.718	13.522
	keto	−389,459.916	−5.812	−389,463.837	–	4.026
chloroform	enol 1	−389,465.001	−9.842	−389,472.953	3.033	79.163
	enol 2	−389,464.343	−9.695	−389,472.149	2.228	20.363
	keto	−389,459.916	−11.895	−389,469.920	–	0.474

^1^ Standard Gibbs free energy in the gas phase. ^2^ Standard Gibbs free energy of solvation. ^3^ Standard Gibbs free energy in the solution phase; $G_{\mathrm{sol}}^{*}$ = $G_{\mathrm{gas}}^{\circ }$ + $∆G_{S}^{*}$ + $∆G^{\circ \to *}$ ($∆G^{\circ \to *}$ amounts to 1.89 kcal/mol at 298.15 K). ^4^ Difference in standard Gibbs free energies of tautomers in the solution phase; $∆G_{\mathrm{sol}}^{*}$ = $G_{\mathrm{sol}}^{*}\left(\mathrm{keto}\right)$ − $G_{\mathrm{sol}}^{*}\left(\mathrm{enol}\;1\;\mathrm{or}\;\mathrm{enol}\;2\right)$. ^5^ Boltzmann distribution in %; calculated using $G_{\mathrm{sol}}^{*}$ at T = 298.15 K.

## Data Availability

The original contributions presented in this study are included in this article/Supplementary Materials. Further inquiries can be directed to the corresponding authors.

## Supplementary Materials

The following supporting information can be downloaded at: https://www.mdpi.com/article/10.3390/foods14132303/s1, Additional information on analytical instruments; Table S1: Standard Gibbs free energies of solvation of 3-oxododecanal tautomers with different functionals and basis sets in chloroform.

## Abbreviations and Nomenclature

The following abbreviations and trivial compound names are used in this manuscript:

aSAFE	Automated solvent-assisted flavor evaporation
borneol	1,7,7-trimethylbicyclo[2.2.1]heptan-2-ol
bornyl acetate	1,7,7-trimethylbicyclo[2.2.1]heptan-2-yl acetate
cAEDA	Comparative aroma extract dilution analysis
(*R*)-carvone	(5*R*)-2-methyl-5-(prop-1-en-2-yl)cyclohex-2-en-1-one
CI	Chemical ionization
(*E*)-*β*-damascenone	(2*E*)-1-(2,6,6-trimethylcyclohexa-1,3-dien-1-yl)but-2-en-1-one
*γ*-decalactone	5-hexyloxolan-2-one
EI	Electron ionization
*trans*-4,5-epoxy-(2*E*)-dec-2-enal	(2*E*)-3-[(2*R*,3*R*)/(2*S*,3*S*)-3-pentyloxiran-2-yl]prop-2-enal
eugenol	2-methoxy-4-(prop-2-en-1-yl)phenol
FD	Flavor dilution
FFAP	Free fatty acid phase
FID	Flame ionization detector
GC	Gas chromatography
GC–O	Gas chromatography–olfactometry
geraniol	(2*E*)-3,7-dimethylocta-2,6-dien-1-ol
geranyl acetate	(2*E*)-3,7-dimethylocta-2,6-dien-1-yl acetate
HPLC	High-performance liquid chromatography
HR	High resolution
HS	Headspace
i.d.	Inner diameter
3-isobutyl-2-methoxypyrazine	2-methoxy-3-(2-methylpropyl)pyrazine
*trans*-isoeugenol	2-methoxy-4-[(1*E*)-prop-1-en-1-yl]phenol
3-isopropyl-2-methoxypyrazine	2-methoxy-3-(propan-2-yl)pyrazine
limonene	1-methyl-4-(prop-1-en-2-yl)cyclohex-1-ene
linalool	3,7-dimethylocta-1,6-dien-3-ol
MS	Mass spectrometry
myrcene	7-methyl-3-methylideneocta-1,6-diene
NMR	Nuclear magnetic resonance
(*Z*)-*β*-ocimene	(3*Z*)-3,7-dimethylocta-1,3,6-triene
*β*-phellandrene	3-methylidene-6-(propan-2-yl)cyclohex-1-ene
*α*-pinene	2,6,6-trimethylbicyclo[3.1.1]hept-2-ene
*β*-pinene	6,6-dimethyl-2-methylidenebicyclo[3.1.1]heptane
RI	Retention index
SDE	Simultaneous distillation extraction
sotolon	3-hydroxy-4,5-dimethylfuran-2(5*H*)-one
SPME	Solid phase microextraction
*γ*-terpinene	1-methyl-4-(propan-2-yl)cyclohexa-1,4-diene
terpinen-4-ol	4-methyl-1-(propan-2-yl)cyclohex-3-en-1-ol
TOF	Time-of-flight
vanillin	4-hydroxy-3-methoxybenzaldehyde

## References

[1] Fu J., Dai L., Lin Z., Lu H. (2013). *Houttuynia cordata* Thunb: A review of phytochemistry and pharmacology and quality control. Chin. Med..

[2] Pradhan S., Rituparna S., Dehury H., Dhall M., Singh Y.D. (2023). Nutritional profile and pharmacological aspect of *Houttuynia cordata* Thunb. and their therapeutic applications. Pharmacol. Res.-Mod. Chin. Med..

[3] Khamsan S., Intecha N., Inpeng P., Wongwan W., Phintakul S., Jitmanee C. (2020). Chemical compositions and anticancer activity of essential oil from *Houttuynia cordata* Thunb. NU. Int. J. Sci..

[4] Gupta S., Bharalee R. (2021). Genetic diversity and population structure of a medicinal herb *Houttuynia cordata* Thunb. of north-east India. Plant Mol. Biol. Rep..

[5] Xu Y.W., Liu L., Zhao D., Zou Y.T., Zeng J.W., Wu W. (2011). Aliphatic aldehyde rich volatile constituents of *Houttuyania cordata* from southwest China. J. Med. Plant Res..

[6] Wang Q., Li Z., Feng X., Li X., Wang D., Sun G., Peng H. (2020). Vegetable *Houttuynia cordata* Thunb. as an important human mercury exposure route in Kaiyang county, Guizhou province, SW China. Ecotoxicol. Environ. Saf..

[7] Wang Q., Li Z., Feng X., Wang A., Li X., Wang D., Fan L. (2021). Mercury accumulation in vegetable *Houttuynia cordata* Thunb. from two different geological areas in southwest China and implications for human consumption. Sci. Rep..

[8] Talukdar R., Wary S., Mili C., Roy S., Tayung K. (2020). Antimicrobial secondary metabolites obtained from endophytic fungi inhabiting healthy leaf tissues of *Houttuynia cordata* Thunb., an ethnomedicinal plant of northeast India. J. Appl. Pharm. Sci..

[9] Lu H., Wu X., Liang Y., Zhang J. (2006). Variation in chemical composition and antibacterial activities of essential oils from two species of *Houttuynia* THUNB. Chem. Pharm. Bull..

[10] Chen J., Wang W., Shi C., Wan J., Deng L., Fang J. (2014). Determination of four volatile compounds with anti-inflammatory biological activity in *Houttuynia chordata* Thunb. by gas chromatography and gas chromatography-mass spectrometry. Anal. Lett..

[11] Lee S.G., Kang H. (2019). Ameliorative effect of *Houttuynia cordata* Thunb (Saururaceae) leaf extract in loperamide-induced constipation in rats. Trop. J. Pharm. Res..

[12] Yang L., Jiang J.G. (2009). Bioactive components and functional properties of *Hottuynia cordata* and its applications. Pharm. Biol..

[13] Chiow K.H., Phoon M.C., Putti T., Tan B.K.H., Chow V.T. (2016). Evaluation of antiviral activities of *Houttuynia cordata* Thunb. extract, quercetin, quercetrin and cinanserin on murine coronavirus and dengue virus infection. Asian Pac. J. Trop. Med..

[14] Verma R.S., Joshi N., Padalia R.C., Singh V.R., Goswami P., Kumar A., Iqbal H., Verma R.K., Chanda D., Chauhan A. (2017). Chemical composition and allelopathic, antibacterial, antifungal, and antiacetylcholinesterase activity of fish-mint (*Houttuynia cordata* Thunb.) from India. Chem. Biodivers..

[15] Yang L., Ji W., Zhong H., Wang L., Zhu X., Zhu J. (2019). Anti-tumor effect of volatile oil from *Houttuynia cordata* Thunb. on HepG2 cells and HepG2 tumor-bearing mice. RSC Adv..

[16] Ju L., Zhang J., Wang F., Zhu D., Pei T., He Z., Han Z., Wang M., Ma Y., Xiao W. (2021). Chemical profiling of *Houttuynia cordata* Thunb. by UPLC-Q-TOF-MS and analysis of its antioxidant activity in C_2_C_12_ cells. J. Pharm. Biomed. Anal..

[17] Wu Z., Deng X., Hu Q., Xiao X., Jiang J., Ma X., Wu M. (2021). *Houttuynia cordata* Thunb: An ethnopharmacological review. Front. Pharmacol..

[18] Lin C.H., Chao L.K., Lin L.Y., Wu C.S., Chu L.P., Huang C.H., Chen H.C. (2022). Analysis of volatile compounds from different parts of *Houttuynia cordata* Thunb. Mol..

[19] Yang Z., Luo S., Ma J., Wu D., Hong L., Yu Z. (2016). GC-MS analyses of the volatiles of *Houttuynia cordata* Thunb. Pak. J. Pharm. Sci..

[20] Asakawa Y., Tomiyama K., Sakurai K., Kawakami Y., Yaguchi Y. (2017). Volatile compounds from the different organs of *Houttuynia cordata* and *Litsea cubeba* (*L. citriodora*). J. Oleo Sci..

[21] Liang M., Qi M., Zhang C., Zhou S., Fu R., Huang J. (2005). Gas chromatography-mass spectrometry analysis of volatile compounds from *Houttuynia cordata* Thunb after extraction by solid-phase microextraction, flash evaporation and steam distillation. Anal. Chim. Acta.

[22] Qi S., Zha L., Peng Y., Luo W., Chen K., Li X., Huang D., Yin D. (2022). Quality and metabolomics analysis of *Houttuynia cordata* based on HS-SPME/GC-MS. Mol..

[23] Ch M.I., Wen Y.F., Cheng Y. (2007). Gas chromatographic/mass spectrometric analysis of the essential oil of *Houttuynia cordata* Thunb by using on-column methylation with tetramethylammonium acetate. J. AOAC Int..

[24] Pan X., Li H., Chen D., Zheng J., Yin L., Zou J., Zhang Y., Deng K., Xiao M., Meng L. (2021). Comparison of essential oils of *Houttuynia cordata* Thunb. from different processing methods and harvest seasons based on GC-MS and chemometric analysis. Int. J. Anal. Chem..

[25] Meng J., Leung K.S., Jiang Z., Dong X., Zhao Z. (2005). Establishment of GC-MS fingerprint of fresh *Houttuynia cordata*. Chem. Pharm. Bull..

[26] Kwon H.D., Cha I.H., Lee W.K., Song J.H., Park I.H. (1996). Antibacterial activity of volatile flavor components from *Houttuynia cordata* Thunb. J. Food Sci. Nutr..

[27] Zhou J., Fan Q., Zhang Y., Castillo R., Xiao M., Liu H., Zhu Z., Liu Y., Yang Y., Zhou Y. (2021). Novel mathematical model for the assessment of similarity of chromatographic fingerprints of volatile oil from *Houttuynia cordata*. Pharmacogn. Mag..

[28] Schlumpberger P., Stübner C.A., Steinhaus M. (2022). Development and evaluation of an automated solvent-assisted flavour evaporation (aSAFE). Eur. Food Res. Technol..

[29] Steinhaus M., Tranchida P.Q. (2019). Gas chromatography-olfactometry: Principles, practical aspects and applications in food analysis. Advanced Gas Chromatography in Food Analysis.

[30] Kreissl J., Schieberle P. (2017). Characterization of aroma-active compounds in Italian tomatoes with emphasis on new odorants. J. Agric. Food Chem..

[31] Zhao Z., Jiang X., Wang L., Wei Z. (1979). Studies on antimicrobial and antiviral compounds—Synthesis of derivatives of decanoyl acetaldehyde. Acta Pharm. Sin..

[32] Guth H., Grosch W. (1989). 3-Methylnonane-2,4-dione—An intense odour compound formed during flavour reversion of soya-bean oil. Lipid/Fett.

[33] Bemelmanns J.M.H. (1979). Review of isolation and concentration techniques. Progress in Flavour Research, Land, D.G., Nursten, H.E., Eds..

[34] Zhao Y., Truhlar D.G. (2007). The M06 suite of density functionals for main group thermochemistry, thermochemical kinetics, noncovalent interactions, excited states, and transition elements: Two new functionals and systematic testing of four M06-class functionals and 12 other functionals. Theor. Chem. Acc..

[35] Pritchard B.P., Altarawy D., Didier B., Gibson T.D., Windus T.L. (2019). New basis set exchange: An open, up-to-date resource for the molecular sciences community. J. Chem. Inf. Model..

[36] Lu T., Chen Q. (2021). Shermo: A general code for calculating molecular thermochemistry properties. Comput. Theor. Chem..

[37] Marenich A.V., Cramer C.J., Truhlar D.G. (2009). Universal solvation model based on solute electron density and on a continuum model of the solvent defined by the bulk dielectric constant and atomic surface tensions. J. Phys. Chem. B.

[38] Hatanaka A. (1993). The biogeneration of green odour by green leaves. Phytochemistry.

[39] Blee E. (1998). Phytooxylipins and plant defense reactions. Prog. Lipid Res..

[40] Kreissl J., Mall V., Steinhaus P., Steinhaus M. (2022). Leibniz-LSB@TUM Odorant Database.

[41] Xue Q., Wang Z., Yin H. (2012). Highly efficient extraction of decanoyl acetaldehyde in *Houttuynia cordata* thunb under steam distillation and its mass spectrometric analysis. J. Anal. Sci..

[42] Liu Y., Yang Y., Wang B., Wang R., Pang J., Jiang Y., Liu Y. (2021). Development and verification of a precolumn derivatization LC-MS/MS method for the pharmacokinetic study of houttuynine of *Houttuynia* essential oil. Mol..

[43] Masur M., Griitzmacher H., Munster H., Budzikiewicz H. (1987). Mass spectrometric fragmentation of the tautomers of 1,3-diketones—A gas chromatographic/mass spectrometric study. Org. Mass Spectrom..

[44] Choudhury B.H., Baruah A.M., Sarmah T.C., Baishya S. (2017). Nutritional and antinutritional composition of twenty five indigenous leafy vegetables of Jorhat district of Assam state, India. Asian J. Chem..

[45] Nowroozi A., Jalbout A.F., Roohi H., Khalilinia E., Sadeghi M., Leon de A., Raissi H. (2009). Hydrogen bonding in acetylacetaldehyde: Theoretical insights from the theory of atoms in molecules. Int. J. Quantum Chem..

