# Supplementary material for: Characterization of the Major Odor-Active Compounds in Fresh Rhizomes and Leaves of Houttuynia cordata by Comparative Aroma Extract Dilution Analysis
Source: Foods. 2025 Jun 28;14(13):2303. doi: 10.3390/foods14132303 (PMC12249027; doi:10.3390/foods14132303)
Supplement: Supplementary file 1 [file foods-14-02303-s001.zip › foods-3707751-supplementary.pdf]

Supplementary Materials

Characterization of the Major Odor-Active Compounds in Fresh Rhizomes and Leaves of *Houttuynia cordata* by Comparative Aroma Extract Dilution Analysis

Zhenli Xu,[1,2] Jing Liu,[3] Johanna Kreissl,[1] Claudia Oellig,[2] Walter Vetter,[2] Martin Steinhaus,[1,*] and Stephanie Frank[1,*]

[1] Leibniz Institute for Food Systems Biology at the Technical University of Munich (Leibniz-LSB@TUM), Lise-Meitner-Straße 34, 85354 Freising, Germany
[2] Institute of Food Chemistry, University of Hohenheim, Garbenstraße 28, 70599 Stuttgart, Germany
[3] Institute of Theoretical Chemistry, Ulm University, Albert-Einstein-Allee 11, 89081 Ulm, Germany

*E-mail: m.steinhaus.leibniz-lsb@tum.de, s.frank.leibniz-lsb@tum.de

Overview

Additional Information on Analytical Instruments

GC–O/FID Instrument

GC–MS Instrument

Heart-Cut GC–GC–HRMS Instrument

Comprehensive Two-Dimensional GC×GC–MS Instrument

Preparative HPLC

NMR Spectroscopy

Additional Table

Table S1. Standard Gibbs Free Energies of Solvation of 3-Oxododecanal Tautomers with Different Functionals and Basis Sets in Chloroform

GC–O/FID Instrument

A Trace Gas Chromatograph Ultra (Thermo Fisher Scientific; Dreieich, Germany) was equipped with a cold on-column injector, a flame ionization detector (FID), and a sniffing-port custom-made from aluminum as detailed in *J. Agric. Food Chem*. **2008**, *56*, 4120–4127. The column was either a DB-FFAP column, 30 m × 0.32 mm i.d., 0.25 µm film thickness (Agilent; Waldbronn, Germany) or a DB-5 column, 30 m × 0.32 mm i.d., 0.25 µm film thickness (Agilent). The carrier gas was helium at 65 kPa (DB-FFAP) and 58 kPa (DB-5) constant pressure. The injection volume was 1 µL or 2 µL. The initial oven temperature was 40 °C for 2 min. Then the temperature was ramped at 6 °C/min to 230 °C (DB-FFAP) or to 240 °C (DB-5), which was held for 5 min. A Y-shaped glass splitter connected the end of the column with two uncoated but deactivated fused silica capillaries, each 50 cm × 0.25 mm i.d., which delivered the column effluent in two equal parts to the FID (250 °C base temperature) and the sniffing port (230 °C base temperature), respectively. For GC–O analysis, a trained assessor placed the nose directly above the sniffing port and evaluated the effluent. Whenever an odor was detected, the position, as well as the odor quality, were marked in the FID chromatogram. For each odorant, a retention index was calculated by linear interpolation from its retention time and the retention times of adjacent *n*-alkanes as detailed in *J. Chromatogr. A*. **1963**, *11*, 463–471.

GC–MS Instrument

A 7890B gas chromatograph (Agilent) was equipped with a GC 80 autosampler and a multimode injector. The column was either a DB-FFAP column, 30 m × 0.25 mm i.d., 0.25 µm film thickness (Agilent) or a DB-5 column, 30 m × 0.25 mm i.d., 0.25 µm film thickness (Agilent). The carrier gas was helium at 1.0 mL/min constant flow. The injection volume was 1 µL or 2 µL. The oven temperature programs were identical to those detailed in the GC–O/FID instrument section. The GC was connected to a Saturn 240 ion trap mass spectrometer (Agilent) operated in the electron ionization (EI) mode at 70 eV and a scan range of *m/z* 40–250 or the chemical ionization (CI) mode with methanol as reagent gas and a scan range of *m/z* 60–250. Data were analyzed with the MS Workstation software (Agilent).

Heart-Cut GC–GC–HRMS Instrument

A Trace 1310 gas chromatograph (Thermo Fisher Scientific) was equipped with a TriPlus RSH autosampler, a programmed temperature vaporizing (PTV) injector, an FID (250 °C base temperature), and a custom-made sniffing port (cf. *J. Agric. Food Chem*. **2008**, *56*, 4120–4127; 230 °C base temperature). The column was a DB-FFAP column, 30 m × 0.25 mm i.d., 0.25 µm film thickness (Agilent). The carrier gas was helium at 1.0 mL/min constant flow. The injection volume was 1 µL or 2 µL. The initial oven temperature was 40 °C for 2 min. Then, the temperature was ramped at 6 °C/min to 230 °C, which was held for 5 min. The end of the column was connected to a Deans switch (S+H Analytik; Mönchengladbach, Germany) used for heart-cutting. The Deans switch directed the eluate of the column via uncoated but deactivated fused silica capillaries (0.1 mm i.d.) time-programmed either simultaneously to the FID and the sniffing port used as monitor detectors or to a second GC column, which was a DB-1701 column, 30 m × 0.25 mm i.d., 0.25 µm film thickness (Agilent). This column was installed in a second Trace 1310 gas chromatograph. The capillary to the second column first passed through a heated (250 °C) hose connecting the two gas chromatographs and then through a liquid nitrogen-cooled trap used to refocus the heart-cut. The initial temperature of the second oven was 40 °C for 2 min. Then the temperature was ramped at 6 °C/min to 240 °C, which was held for 5 min. The end of the second column was connected to a Q Exactive GC orbitrap mass spectrometer (Thermo Fisher Scientific) operated in the high-resolution EI mode at 70 eV and a scan range of *m/z* 40–300 or the high-resolution CI mode with isobutane as reagent gas and a scan range of *m/z* 95–200. Data were analyzed with the Xcalibur software (Thermo Fisher Scientific).

Comprehensive Two-Dimensional GC×GC–MS Instrument

A 6890 Plus gas chromatograph (Agilent) was equipped with a GC PAL autosampler (CTC Analytics; Zwingen, Switzerland) and a CIS 4 injector (Gerstel; Mülheim an der Ruhr, Germany). The column in the first dimension was a DB-FFAP column, 30 m × 0.25 mm i.d., 0.25 µm film thickness (Agilent). The carrier gas was helium at 2.0 mL/min constant flow. The injection volume was 2 µL. The initial oven temperature was 40 °C for 2 min. Then the temperature was ramped at 6 °C/min to 230 °C, which was held for 5 min. The end of the first column was connected via a liquid nitrogen-cooled dual-stage quad-jet modulator (Leco; Mönchengladbach, Germany) to a DB-5 column, 3 m × 0.15 mm i.d., 0.30 µm film thickness (Agilent) inside the secondary oven, which was mounted inside the primary GC oven. The modulation time was 4 s. The initial temperature of the second oven was 70 °C for 2 min. Then the temperature was ramped at 6 °C/min to 250 °C, which was held for 5 min. The end of the second column was connected to a Pegasus III TOF mass spectrometer (Leco) operated in the EI mode at 70 eV with a scan range of m/z 35–350 and a scan rate of 100 spectra/s. Data were analyzed with the GC Image software (GC Image; Lincoln, Nebraska, USA).

Preparative HPLC

An AZURA HPLC system (Knauer; Berlin, Germany) was equipped with an autosampler AZURA 6.1L, a binary high-pressure pump system P6.1L HPG, a UV detector MWD 2.1L used at 270 nm, and a fraction collector LABOCOL Vario 4000. The column was a preparative Kinetex C18 column, 150 mm × 10.0 mm, 5 µm particle size, with security guard cartridge C18, 4 mm × 3.0 mm (Phenomenex; Aschaffenburg, Germany). The injection volume was 50 µL. A gradient of 0.1% formic acid in water/acetonitrile (40/60; v/v) as solvent A and acetonitrile as solvent B was used (3 mL/min): 0 min, 0% B; 0.5 min, 0% B; 25 min, 100% B; 30 min, 100% B; 35 min, 0% B; and 40 min, 0% B. Data were analyzed with the PurityChrome software (Knauer).

NMR Spectroscopy

NMR spectra were acquired on an Avance-III 400 MHz NMR spectrometer (Bruker; Rheinstetten, Germany) with frequencies of ^{1}H (400 MHz) and ^{13}C (100 MHz) at 25 °C. All signals were referenced to the solvent signals at δ = 7.24 ppm. Data were processed and analyzed with the TopSpin software 3.7 (Bruker).

Table S1. Standard Gibbs Free Energies of Solvation of 3-Oxododecanal Tautomers with Different Functionals and Basis Sets in Chloroform

functional and basis set[a]	ΔG_S^* (kcal/mol)		
	enol 1	enol 2	keto
M05[1]/6-311++G(2d,2p)[2,3]	−10.033	−10.007	−12.823
M05[1]/6-31G(d)[4–6]	−9.391	−9.268	−11.506
M05[1]/6-31+G(d,p)[2,4–6]	−10.264	−10.226	−13.147
M05[1]/cc-pVTZ[7]	−9.526	−9.464	−12.026
M052X[8]/6-311++G(2d,2p)[2,3]	−10.520	−10.486	−13.086
M052X[8]/6-31G(d)[4–6]	−9.739	−9.596	−11.673
M052X[8]/6-31+G(d,p)[2,4–6]	−10.564	−10.489	−13.152
M052X[8]/cc-pVTZ[7]	−10.256	−10.205	−12.769
M06[9]/6-311++G(2d,2p)[2,3]	−9.883	−9.817	−12.569
M06[9]/6-31G(d)[4–6]	−9.357	−9.193	−11.427
M06[9]/6-31+G(d,p)[2,4–6]	−10.061	−9.967	−12.825
M06[9]/cc-pVTZ[7]	−9.458	−9.361	−11.913
M062X[9]/6-311G(d,p)[10]	−9.842	−9.695	−11.895
M062X[9]/6-311++G(2d,2p)[2,3]	−10.242	−10.184	−12.608
M062X[9]/6-31G(d)[4–6]	−9.476	−9.317	−11.268
M062X[9]/6-31+G(d,p)[2,4–6]	−10.217	−10.118	−12.612
M062X[9]/cc-pVTZ[7]	−9.965	−9.885	−12.298
PBEh1PBE[11]/6-311++G(2d,2p)[2,3]	−10.080	−10.033	−12.547
PBEh1PBE[11]/6-31G(d)[4–6]	−9.458	−9.309	−11.305
PBEh1PBE[11]/6-31+G(d,p)[2,4–6]	−10.202	−10.124	−12.717
PBEh1PBE[11]/cc-pVTZ[7]	−9.743	−9.668	−12.066
wB97XD[12]/6-311++G(2d,2p)[2,3]	−10.120	−10.066	−12.656
wB97XD[12]/6-31G(d)[4–6]	−9.520	−9.369	−11.458
wB97XD[12]/6-31+G(d,p)[2,4–6]	−10.252	−10.168	−12.856
wB97XD[12]/cc-pVTZ[7]	−9.749	−9.670	−12.129

[a]Functionals and basis sets as detailed in 1. *J. Chem. Theory Comput.* **2006**, *2*, 364–382; 2. *J. Comput. Chem.* **1983**, *4*, 294–301; 3. *J. Chem. Phys.* **1980**, *72*, 650–654; 4. *J. Chem. Phys.* **1971**, *54*, 724–728; 5. *Theor. Chim. Acta* **1973**, *28*, 213–222; 6. *J. Chem. Phys.* **1972**, *56*, 2257–2261; 7. *J. Chem. Phys.* **1989**, *90*, 1007–1023; 8. *J. Chem. Phys.* **2005**, *123*, 161103; 9. *Theor. Chem. Acc.* **2007**, *120*, 215–241; 10. *J. Chem. Inf. Model.* **2019**, *59*, 4814–4820; 11. *J. Chem. Phys.* **1998**, *109*, 3313–3320; 12. *Phys. Chem. Chem. Phys.* **2008**, *10*, 6615–6620.
